# Ergosterol concentration and variability in genotype-by-pathogen interaction for grain mold resistance in sorghum

**DOI:** 10.1007/s00425-014-2081-7

**Published:** 2014-05-11

**Authors:** Leo T. Mpofu, Neal W. McLaren

**Affiliations:** 1 Present Address: ZSAES, P. Bag 7006, Chiredzi, Zimbabwe; 2 Matopos Research Station, P. Bag K5137, Bulawayo, Zimbabwe; 3 Plant Sciences Plantwetenskappe, P O Box 339, Bloemfontein, 9300 Republic of South Africa

**Keywords:** Ergosterol, Grain mold, Resistance

## Abstract

A lack of understanding of host-by-pathogen relations can hinder the success of breeding for resistance to a major disease. Fungal strain pathogenicity has to be understood from the virulence it can cause on susceptible genotypes and host resistance indicates which genotypes have resistance genes. Where the two worlds meet lies the place where researchers match the prevalent pathogen in the area of production with resistant varieties. This paper uses ergosterol concentration analysis as a measure of fungal biomass accumulation to assess levels of resistance in host genotypes. 11 sorghum genotypes were inoculated with 5 strains of fungi that are known to be associated with grain mold disease of sorghum. The resulting interaction was analyzed using GGE Biplot analysis and Cluster analysis which showed that none of the genotypes were resistant to *Phoma sorghina* and *Curvularia lunata*. Three genotypes were resistant to *Fusarium thapsinum*. One fungal strain (*Alternaria alternata*) does not contribute any significant damage in the grain mold disease. *Fusarium graminearum* causes very little grain mold disease. There was no correlation between the fungal strains. Visual scoring did not correlate with ergosterol accumulation. Resistance to grain mold in sorghum is shown to be due to vertical or specific resistance genes. Sorghum breeders should, therefore, identify predominant fungal strains in their localities and then locate and tag these resistance genes in their germplasm and pyramid them in commercial varieties.

## Introduction

Grain mold is a significant constraint to sorghum (*Sorghum bicolor* (L.) Moench) production and utilization. It is a particular problem in areas where the period between anthesis and harvest coincides with high humidity and warm temperatures. Most of the fungi isolated from molded grain are facultative parasites and the predominant species differ across locations and seasons. The significance of sorghum grain mold has been highlighted in Africa, Asia and the Americas (Frederiksen et al. 1982; ICRISAT 1987). Grain mold reduces yield, nutritional quality, seed viability, kernel weight and market value (Forbes et al. 1992). Grain mold fungi are also responsible for the production of potent mycotoxins and secondary metabolites that are harmful to human and animal health and productivity (Castor and Frederiksen 1980). Fungi in more than 40 genera have been associated with sorghum grain mold (Williams and Rao 1981). Mycoflora analysis of sorghum kernels over the years reveals that some of the most important species include *Fusarium graminearum* Schwabe*, Fusarium thapsinum* Klittich, Leslie, Nelson et Marasas sp. nov. 1996*, Curvularia lunata* (Wakker) Boedijn, *Phoma sorghina* (Sacc.) Boerma et al. and *Alternaria alternata* (Fr.) Keissl. because they are more frequently isolated from molded grain (Williams and Rao 1981; Bandyopadhyay et al. 1991; Esele et al. 1993; Erpelding and Prom 2006).

Grain mold is a result of a complex fungus–host interaction. This interaction needs to be fully investigated and understood before a durable solution to grain mold damage is found. Several publications have referred to a wide range of fungi as the “grain mold complex” fungi simply because at some point they have been associated with moldy grain sorghum. They have, therefore, sought to find a resistance mechanism that would restrict all grain mold fungi simultaneously. Grain characteristics that have been associated with mold resistance include grain hardness, a thin pericarp, a thick surface wax layer, a pigmented testa, a red pericarp, high concentration of tannins and flavan-4-ols, antifungal proteins, grain density and grain integrity, open panicles with long glumes, and plant height (Glueck and Rooney 1980; Mukuru 1992; Esele et al. 1993; Rodriguez-Herrera et al. 1999; Waniska et al. 2001). Most of these traits are qualitative traits. Overall, grain mold resistance is believed to be multigenic with recent estimates indicating a minimum of 4–10 genes controlling resistance in white-grained sorghums (Rodriguez-Herrera et al. 2000).

Before resistance mechanisms can be investigated, it is essential to understand fungal pathogenicity. Somani et al. (1994) reported the presence of strains of *Curvularia lunata* that poses different levels of pathogenicity to sorghum in India. On the other hand, Jardine and Leslie (1992) found no differences in pathogenicity among two mating populations of *Fusarium* spp. Infection and colonization patterns were found to differ for *Fusarium* spp. and *Curvularia lunata* (Castor 1981). These differences may partially explain why resistance to the two pathogens also differs and why the expression of resistance under changing conditions varies. After evaluating several thousand germplasm lines in the International Grain Mold Resistance Screening Program (Indira et al. 1991) noted that no genotype was immune to grain mold. This shows that no single genotype has all the necessary resistance genes. However, recombination breeding (crosses between lines with some resistance and susceptible ones) improved varietal resistance to grain mold. This improvement could be attributed to chance recombination among crosses. Informed crosses could have achieved more genetic gain toward achieving immunity. The little gain observed was achieved using visual grain mold rating during the screening process under natural infection.

Host-by-pathogen interaction in the sorghum grain mold disease complex can present a complicated relationship that many scientists have either taken for granted or struggled to explain, more so when there are many pathogens and genotypes involved. Grain mold in sorghum presents a typical case of a very complex host-by-pathogen interaction. Predominant use of visual scoring for sorghum grain mold damage has led to two major complications: (1) Resistant sorghum varieties being classified as susceptible to grain mold when in reality the fungus is growing on the peripheral layers of the kernel causing very little internal damage. (2) Susceptible varieties being classified as resistant when the internal structures are highly infected with fungal biomass despite a cleaner outward look.

Measurement of ergosterol concentration is a more sensitive method of estimating total (viable and non viable) fungal biomass which considers all fungal growth events that have taken place (Seitz et al. 1977). Chitin has been used to measure fungal growth in maize, soybean (Donald and Mirocha 1977) and wheat (Golubchuk et al. 1960). However, this method was found to be less sensitive compared to ergosterol determination. Determination of ergosterol is a sensitive indicator of fungal invasion in grain. Ergosterol is the predominant sterol component of all fungi (Weete 1974) and it differs significantly from sterols of higher plants. It is, therefore, not a native constituent of grains. The primary role of sterols in nature is as architectural components of membranes (Nes 1974). Ergosterol concentration procedure has been used to distinguish levels of grain mold resistance (Forbes et al. 1989; Jambunathan et al. 1991; Audilakshmi et al. 1999). In principle, the more the ergosterol in a grain the more the damage caused and the more susceptible the genotype affected. Ergosterol measurement provides an indication of the extent of internal mold colonization which is not externally visible. Therefore, a combination of assessment of severity of different fungi (visually or on agar) in a grain sample in conjunction with ergosterol measurement indicates the identity of the fungi and their quantity.

The objectives of this research are: 1) to find out if fungi that have been previously associated with the grain mold complex disease contribute equally to observed damage, 2) to use ergosterol accumulation in sorghum grain as a measure of fungal biomass accumulation to distinguish between resistant and susceptible sorghum varieties, 3) to find out if sorghum genotypes exhibit resistance to specific fungi or to the whole grain mold complex fungi as previously believed.

## Materials and methods

### Genetic material and fungal cultures

In August 2006, a diverse array of 11 sorghum genotypes with different reactions to mold was selected for this program. The 11 genotypes were sourced from a Southern Africa sorghum regional breeding nursery supported by the International Sorghum and Millets Collaborative Research Support Program (INTSORMIL CRSP) of the USAID.

The experiment was conducted in a greenhouse at the University of the Free State in Bloemfontein South Africa. The selected genotypes were classified into three groups R (resistant), I (intermediate), and S (susceptible) (Table 1) based on field grade score (FGS) data from a number of years of prior field evaluations. These data were obtained using a rating system described by (Bandyopadhyay and Mughogho 1988). Ratings were based on a 1–5 scale in which 1 = no visible mold; 2 = 1–10 %; 3 = 11–25 %; 4 = 26–50 %; 5 = more than 50 % of kernels in the panicle molded. Genotypes in the Group (R) are resistant and have a score (*x*) such that *x* ≤ 2. Genotypes in the Group (I) are intermediate resistant and 2 < *x* ≤ 3. Genotypes in the Group (S) are susceptible genotypes and 3 < *x* ≤ 5.

**Table 1:** Pedigree, field grade score (FGS), and level of resistance of 11 sorghum genotypes planted in the greenhouse in 2006 for evaluation for genetic response to grain mold

Genotype #	Pedigree	FGS	Resistance level^a^
1	(87EO366 * WSV387)-HF14	1.25	R
2	(ISCV 1089BF * MACIA)-HF2-CA2-AE	1.5	R
3	(MACIA * DORADO)-HD2—CA3	1.75	R
4	(MACIA * TAMU428)-LL9	3	I
5	(Segaolane*WM#322)-CG1-BGBK-CCBK	2.5	I
6	(SV1*Sima/IS23250)-LG15-CG1-BG2-BGBK	2.5	I
7	(90EO328*CE151)-LA37	1.25	R
8	Kuyuma	4.25	S
9	R.9645_(RTx430*Sureno)-B12	4.5	S
10	R.9732_(ADN55*Tx430)-B10	4	S
11	SRN39_Striga Res.	3	I

^a^Level: R, resistant (*x* ≤ 2); I, intermediate resistant (2 < *x* ≤ 3); S, susceptible to grain mold 3 < *x* ≤ 5

Plants were maintained at 25–30 °C with regular irrigation and fertilization. Seed from each line was sown into a steam sterilized soil:peat (3:1) mix in 25-cm diameter pots and thinned to two plants per pot after emergence. There was one pot per replication. A split-plot design with three replications was used with fungus as the main treatment (whole-plot) and genotypes as the sub-plots within treatment. Genotypes and fungi were fixed. Panicles were visually scored for grain mold severity and harvested 50 days after anthesis and were evaluated for ergosterol content.

### Sources of isolates

Isolates of *F. graminearum* and *F. thapsinum* were obtained from grain collected at Potchefstroom during 2005–2006 seasons and maintained in a culture collection at the University of the Free State. Isolates of *Curvularia lunata, Phoma sorghina* and *Alternaria alternata* were obtained from bulk sorghum grain sampled from a local brewing company in the same season. Grain was surface sterilized in 1 % sodium hypochlorite solution for 3 min followed by three rinses in sterile distilled water. The grain was dried on sterile blotting paper and 50–100 seeds were plated onto malt extract agar (MEA) medium (Biolab Diagnostics SA (Pty) Ltd) to which streptomycin (Caps Pharmaceuticals SA (Pty) Ltd/(Edms) Bpk was added at a rate of 0.3 ml per liter of medium. Individual fungal colonies from grains were transferred to half-strength potato dextrose agar (PDA) (Biolab) to assess colony morphology. Single-spore isolates obtained following serial dilution of spores collected from colonies growing on ½ PDA were cultured on full-strength PDA to bulk inoculum.

### Inoculum production

Conidial suspensions of *F. graminearum, F. thapsinum, Curvularia lunata, Phoma sorghina* and *Alternaria alternata* were prepared by plating single colony agar plugs onto a culture plate and harvesting conidia by scraping the colonized agar plate with a flame sterilized bacterial spreader. The solution was filtered through autoclaved cheese cloth to remove mycelial fragments. Conidial suspensions were calibrated to 1 × 10^6^ conidia per ml of sterile water with a hemocytometer.

### Plant inoculation

Individual genotypes were inoculated with spores of the five fungi and the treatments were replicated three times. Heads sprayed with sterile water served as a control. Panicles were inoculated at grain milk dough stage. Inoculum was sprayed at all angles on to the panicles until runoff. Panicles were immediately covered with a plastic bag for 7 days to maintain high relative humidity and promote initial infection and colonization. The incidence of grain mold and its damage was scored visually as described above at maturity. The identity of grain mold fungi in the greenhouse was confirmed by confirmatory re-isolations in the lab.

### Ergosterol extraction and determination

At maturity, grain was harvested from each treatment replicate and ergosterol was determined according to the method of Seitz et al. (1977) as modified by Jambunathan et al. (1991). A 10 g sorghum grain sample from each panicle was ground using a laboratory mill and sieved through a 0.4 mm screen. Ergosterol was then extracted from the sample with 50 ml of methanol (MeOH) by vigorously mixing with a magnetic stirrer in a 100 ml beaker for 30 min. The mixture was allowed to settle and 25 ml of clean extract was decanted and added to a screw capped test tube containing 3 g of potassium hydroxide (KOH). The mixture was thoroughly agitated on a vortex mixer to dissolve KOH. *n*-Hexane (10 ml) was added and the mixture was incubated at 75 °C in a water bath for 30 min and allowed to cool to room temperature. Distilled water (5 ml) was added, and after mixing thoroughly, the solution was allowed to cool to room temperature. The upper hexane layer was removed with a syringe and transferred to a test tube. Hexane (10 ml) was added to the remaining aliquot in the screw capped test tube and mixed thoroughly and the upper hexane layer was again removed and pooled with the earlier aliquot. The procedure was repeated one more time. The three pooled hexane extracts in the test tube were evaporated to dryness in a hot water bath maintained at 75 °C. The residue was re-dissolved in 5 ml of methanol (HPLC grade) and filtered through a 0.45 μm filter (Millex-HV, Millipore Corp., Bedford, MA). A 2 ml aliquot of the filtrate was maintained in a −80 °C freezer for ergosterol determination.

Ergosterol content in the filtrate was determined using a Shimadzu DGU-20A5 Prominence Degasser high-performance liquid chromatography (HPLC) with auto injector SIL-20A. The extract was loaded onto a silica-based reverse-phase column (C18 110 Å 5 μm particle size, 150 × 4.6 mm). The mobile phase was methanol–water (96:4 v/v) at a flow rate of 1.2 ml/min. The column temperature was maintained at 50 °C and the absorbance of eluted ergosterol was detected with a SPD-M20A prominence diode array detector at 282 nm. The standard ergosterol (Sigma) had a retention time of 8.2 min. The area under the graph for all chromatograms was converted to ergosterol concentration in μg/g using the following best fit formula:

1 $$[Y = \, 0. 100 8{{\text{e}}^{ 7 {\text{E}} - 0 8 {\text{AREA}}}}]$$

where *Y* is the ergosterol concentration in μg/g of grain and *AREA* is the area under the chromatogram graph of micro-absorbance units (mAU) versus time (minutes).

### Statistical analysis

Analyses of replicated green house data were done using SAS (SAS Enterprise Guide 2005) and Microsoft Excel. Kolmogorov–Smirnov (K–S) test and Bartlett’s test were conducted prior to data analysis to test for normality and homogeneity of error, respectively (Steel and Torrie 1980). Data were subjected to analysis of variance (ANOVA). Tuckey–Kramer method at the 5 % significance level was used to separate means. Rep*pathogen was used as error term for testing fungi/pathogens while Rep*Genotype was used to test the genotype main effect. Least Square Means (LSMeans) were then used to generate a genotype × pathogen two-way table that was later used in the GGE and cluster analysis. Pearson coefficients were used to test for correlations among the fungi themselves and also to test the relationship between the use of ergosterol concentration versus field grade scores or visual scoring.

GGE biplot analysis software (Yan 2001) was used to explain the significant genotype-by-pathogen (G × P) interaction observed after ANOVA. Both *strain*-*focused* and *genotype*-*focused* biplots (Yan 2001) were used to evaluate fungal strain virulence and host plant resistance, respectively, using equations:

2 $$[{\hat Y_{ij}} - { \propto_i} = {\lambda_1}{\xi_{i1}}{\eta_{1j}} + {\lambda_2}{\xi_{i2}}{\eta_{2j}} + {\varepsilon_{ij}}\quad {\text{strain-focused biplot}}]$$

3 $$[{\hat Z_{ij}} - {\beta_j} = {\lambda_1}{\xi_{i1}}{\eta_{1j}} + {\lambda_2}{\xi_{i2}}{\eta_{2j}} + {\varepsilon_{ij}}\quad {\text{genotype-focused biplot}}]$$

where ${\hat Y_{ij}}$ is the expected ergosterol concentration of genotype *i* (=1–11) to fungal strain *j* (=1–5); ${\hat Z_{ij}}$ is the expected ergosterol concentration of strain *i* (=1–5) to genotype *j* (=1–11); ${ \propto_i}$ is the mean ergosterol content of genotype *i* across all strains; ${\beta_j}$ is the mean ergosterol content of strain *j* across all genotypes; ${\lambda_1}$ and ${\lambda_2}$ are the singular values for PC1 and PC2, respectively; ${\xi_{i1}}$ and ${\eta_{1j}}$ are the PC1 eigenvectors for genotype *i* and strain *j*, respectively; ${\xi_{i2}}$ and ${\eta_{2j}}$ are the PC2 eigenvectors for genotype *i* and strain *j*, respectively; and ${\varepsilon_{ij}}$ is the residue for each genotype–strain combination not explained by PC1 and PC2.

GGE biplot was also used to determine: if the resistance is vertical or horizontal, how to group the genotypes based on their resistance, which genotypes might carry gene(s) for resistance to which fungus, how to group the fungal strains based on their virulence, what is the appropriate strategy for breeding resistance to the pathogens under investigation.

Cluster analysis of SAS (SAS Enterprise Guide 2005) was used on the two-way table. A combination of GGE Biplot analysis and cluster analysis was used to consolidate the grouping of genotypes and that of fungal strains in detail.

## Results

Most of the variation is among fungal strains (65.3 %) than among genotypes (8.1 %) (Table 2). This indicates a need to thoroughly understand grain mold fungi biology and etiology because variation here could explain most of the grain mold damage. The interaction between fungal strains and genotypes accounts for 26.6 % of the variation compared to the 8.1 % for genotypes indicating that different genotypes responded significantly different to infection by grain mold fungi or vice versa, i.e., different fungi caused significantly varying damage to the different genotypes. Variation among genotypes indicates a more vertical nature of resistance to grain mold fungi, i.e., a few genes could be involved in the genotype reaction.

**Table 2:** Analysis of variance for ergosterol concentration of 11 genotypes treated with *Fusarium graminearum, Fusarium thapsinum, Curvularia lunata, Phoma sorghina* and *Alternaria alternata* spores plus a control in the greenhouse

Source	*df*	SS	MS	*F*	% of (G + P + GP)
Model	77	556.44^a^	7.23	8.92**	
Rep	2	0.80	0.40	0.49 ns	
Genotype	10	44.40	4.44	5.39**	8.1
Pathogen	5	355.62	71.12	66.82**	65.3
Gen × pathogen	50	144.97	2.90	3.58**	26.6
Rep × pathogen	10	10.64	1.06	1.31 ns	
Rep × genotype	20	16.47	0.82	1.02 ns	
Error	120	97.24	0.81		
Total	197	653.68			

Dependent variable: ergosterol concentration

*ns* not significant

Significant at ** * P* = 0.01

^a^ *R* ^2^ = 0.88 (adjusted *R* ^2^ = 0.76)

Table 3 indicates that there was no correlation among all fungi except for *Alternaria alternata* with *Fusarium thapsinum*. This indicates that most fungi behaved uniquely, hence, there is need for alternative analyses to see if there are possible groupings. Table 3 also indicates that there was no correlation between visual scoring and ergosterol accumulation in grain for four of the fungi. This observation implies that using visual scoring to characterize genotypes as either resistant or susceptible may not be the most accurate way since it does not relate to fungal biomass inside the grain. Since ergosterol accumulation measures actual fungal biomass, it should be the better of the two methods. The only correlation was negative between FGS and *Alternaria alternata* implying that there could be fungal infection in the absence of visual *Alternaria alternata* growth. Visual scoring could be influenced by distribution of the fungal biomass in grain. If most of the fungal hyphae are peripheral, that could lead to higher visual scores while most of the grain is uninfected. These two methods should not be used concurrently.

**Table 3:** Correlations to test the relationship among the fungal treatments and also to test if visual scoring relates to fungal biomass accumulation inside the grain

	*A. alternata*	*C. lunata*	*P. sorghina*	Control	*F. thapsinum*	*F. graminearum*
*C. lunata*	−0.24 ns					
*P. sorghina*	−0.04 ns	0.006 ns				
Control	0.18 ns	0.27 ns	−0.02 ns			
*F. thapsinum*	0.66*	0.12 ns	−0.16 ns	0.006 ns		
*F. graminearum*	−0.37 ns	0.18 ns	−0.44 ns	0.04 ns	0.11 ns	
FGS	−0.7*	0.21 ns	−0.34 ns	−0.25 ns	−0.38 ns	0.27 ns

Asterisk indicates significance at *p* = 0.05

*ns* not significant

### Ranking and grouping of fungal strains

**Fig. 1:**
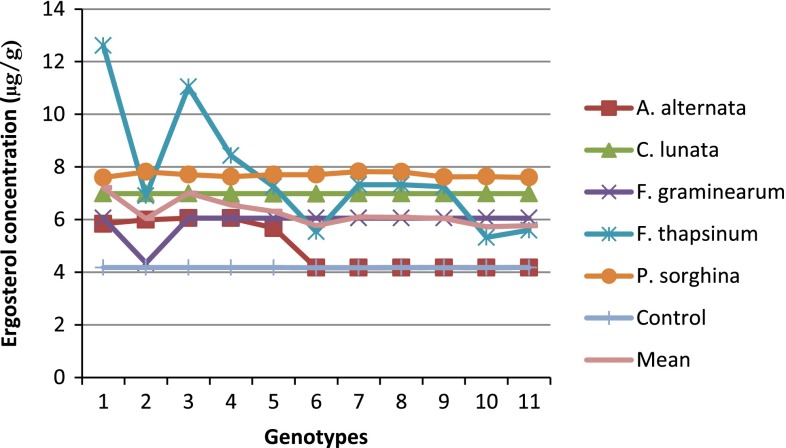
Mean ergosterol concentration across 11 genotypes inoculated with *Fusarium graminearum, Fusarium thapsinum, Curvularia lunata, Phoma* *sorghina*, *Alternaria alternata* and a control in the greenhouse at Bloemfontein, South Africa in 2006

**Fig. 2:**
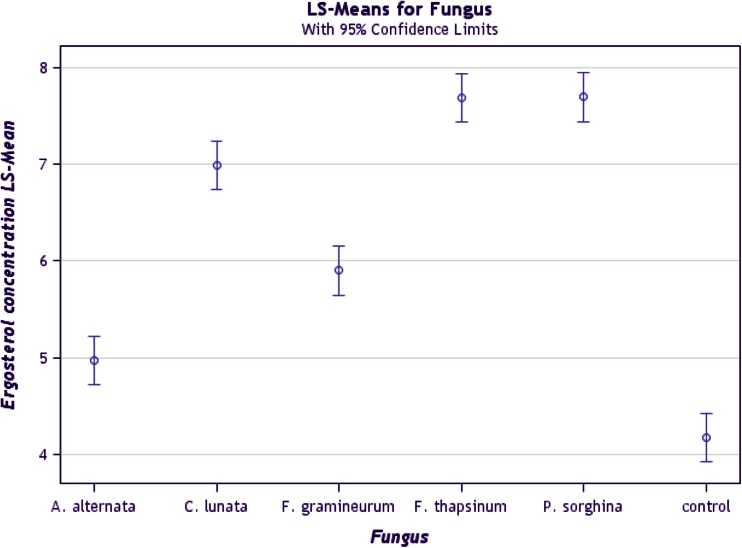
Separation of means for the fungal strains (*Fusarium graminearum, Fusarium thapsinum, Curvularia lunata, Phoma* *sorghina* and *Alternaria alternata*) plus a control over the 11 genotypes

**Fig. 3:**
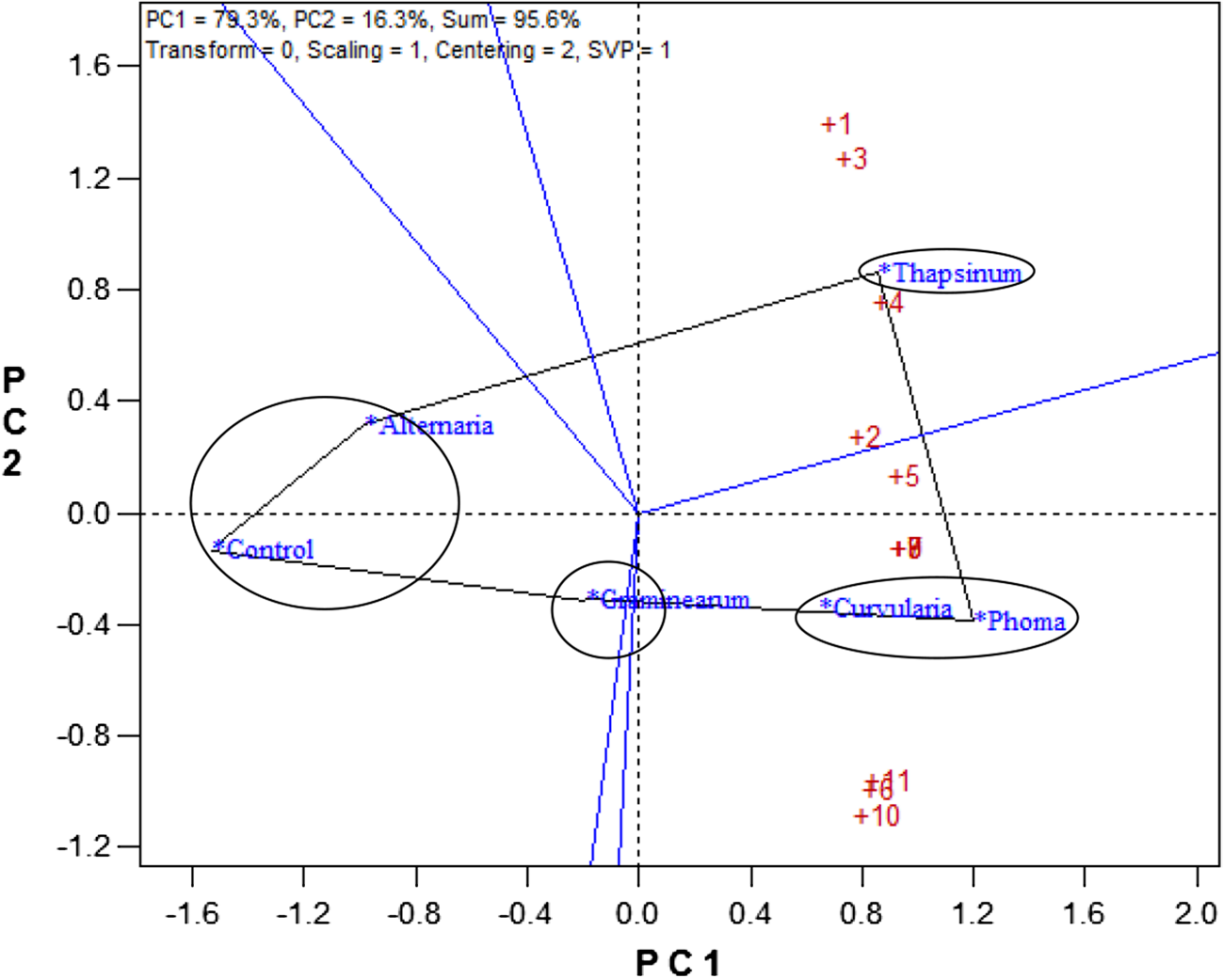
Pathogen strain-focused (genotype-centered) GGE biplot based on mean ergosterol concentration (μg/g of grain) of 11 sorghum varieties inoculated with spores of 5 fungal isolates (*Fusarium graminearum, Fusarium thapsinum, Curvularia lunata, Phoma* *sorghina* and *Alternaria alternata*) plus a control. The fungal isolates were used as entries (*blue*) and genotypes as testers (*red numeric*)

A biplot allows for the analysis of the two-way interaction in a table of (*k*) objects by (*j*) variables such that systematic patterns between rows, between columns, and between rows and columns can be evaluated. It gives “best” representation in low-dimensional space (Kroonenberg 2007). The genotype-by-pathogen strain data were used to calculate the principal component (PC) scores indicated in Table 4. Figure 1 graphically indicates that the control had the lowest levels of ergosterol with all fungal strain varying above that. Figure 2 shows how the fungal biomass mean accumulation separate over all the 11 genotypes. This shows the significance of the differences. The pathogen strain-focused (genotype-centered) GGE biplot shown in Fig. 3 was developed from principal component (PC) scores indicated in Table 4. These scores are calculated using formulae described by Yan (2001). This biplot facilitates identification of isolate groups that are most virulent to each of the genotypes and explains 95.6 % of observed variation. The GGE biplot software draws a polygon by joining isolates located furthest from the biplot origin. Starting from the biplot origin, perpendicular lines are drawn to each side of the polygon, which divides the biplot into sectors. The isolates at the vertices are the most virulent to all genotypes in that sector.

**Table 4:** PC1 and PC2 scores for the five fungal strains (*Fusarium graminearum, Fusarium thapsinum, Curvularia lunata, Phoma* *sorghina* and *Alternaria alternata*) plus a control and 11 genotypes used for generating the strain-focused biplot of Fig. 3

	PC1	PC2
*Entries*	*S*_*j*1_	*S*_*j*2_
*P. sorghina*	1.201	−0.387
*F. thapsinum*	0.855	0.859
*C. lunata*	0.648	−0.34
*F. graminearum*	−0.19	−0.314
*A. alternata*	−0.987	0.314
Control	−1.528	−0.132
*Testers*	*g*_*i*1_	*g*_*i*2_
Genotype 1	0.649	1.39
Genotype 2	0.757	0.273
Genotype 3	0.71	1.272
Genotype 4	0.839	0.751
Genotype 5	0.895	0.13
Genotype 6	0.8	−0.99
Genotype 7	0.903	−0.13
Genotype 8	0.903	−0.128
Genotype 9	0.902	−0.129
Genotype 10	0.773	−1.088
Genotype 11	0.807	−0.959

According to Fig. 3, *Fusarium thapsinum* and *Phoma* *sorghina* are the main grain mold causing fungi among the five because they are at the vertexes. *Fusarium thapsinum* is most virulent to genotypes 1 and 3 because they are in the same sector (confirm with Table 2) while *Phoma* *sorghina* is most virulent to genotypes 6, 10 and 11. On the other hand, genotypes 1 and 3 seem to be resistant to *Phoma* *sorghina* because they are in opposite sectors, but they are not. In fact, none of the genotypes is resistant to *Phoma* *sorghina* because all genotypes have relatively high ergosterol levels when inoculated with *Phoma* *sorghina* spores. *Curvularia lunata* virulence is similar to that of *Phoma* *sorghina*, hence, they are positioned close together in the same sector. *Curvularia lunata* is virulent to all genotypes because all genotypes inoculated with *Curvularia lunata* have high levels (higher than the mean) of ergosterol concentration. Genotypes 6, 10 and 11 on the other hand are resistant to *Fusarium thapsinum* because they are in opposite sectors and their ergosterol levels are lower (even lower than the mean for the strain) relative to other genotypes when inoculated with *Fusarium thapsinum* spores. Susceptibility or resistance of genotypes 2, 4, 5, 7, 8, and 9 is intermediate to both *Fusarium thapsinum* and *Phoma* *sorghina.* Genotypes 1 and 10 are located furthest from the biplot origin (long vectors) and are, therefore, more discriminating than the rest.

**Fig. 4:**
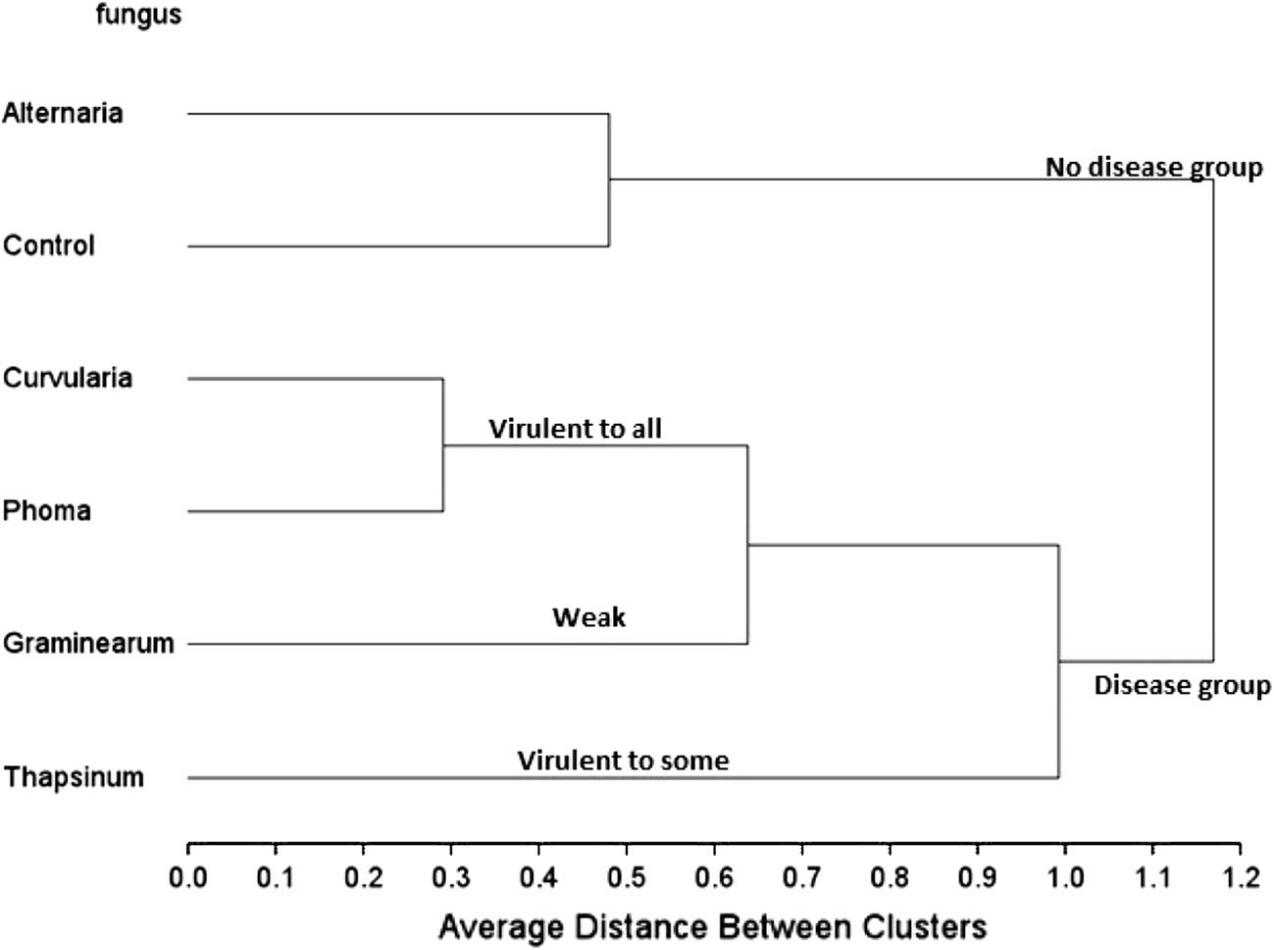
Dendrogram showing clusters of fungal isolate groups (*Fusarium graminearum, Fusarium thapsinum, Curvularia lunata, Phoma* *sorghina* and *Alternaria alternata*) plus a control based on their virulence to the 11 sorghum genotypes

Figure 3 on its own does not show specific grouping of fungal strains adequately as it is based on correlations. The patterns displayed in Figs. 2 and 3 can be better summarized in a dendrogram (Fig. 4) in which clusters of fungal strains are presented. The five fungal isolates were first divided into two large groups, *Alternaria alternata* with the control (no disease caused group) and the other four in another group (disease causing group). This means that *Alternaria alternata* is a superficial grain mold fungus that usually fails to infect and spread into sorghum grain tissue. This is true despite the fact that in some environments *Alternaria alternata* is one of the most frequently isolated species in moldy grain. The other four fungi can be classified into: (1) highly virulent and broad acting fungi, i.e., *Curvularia lunata* and *Phoma* *sorghina*, (2) virulent to specific genotypes, i.e., *Fusarium thapsinum*, and (3) generally virulent but causing less disease, i.e., *Fusarium graminearum*.

**Fig. 5:**
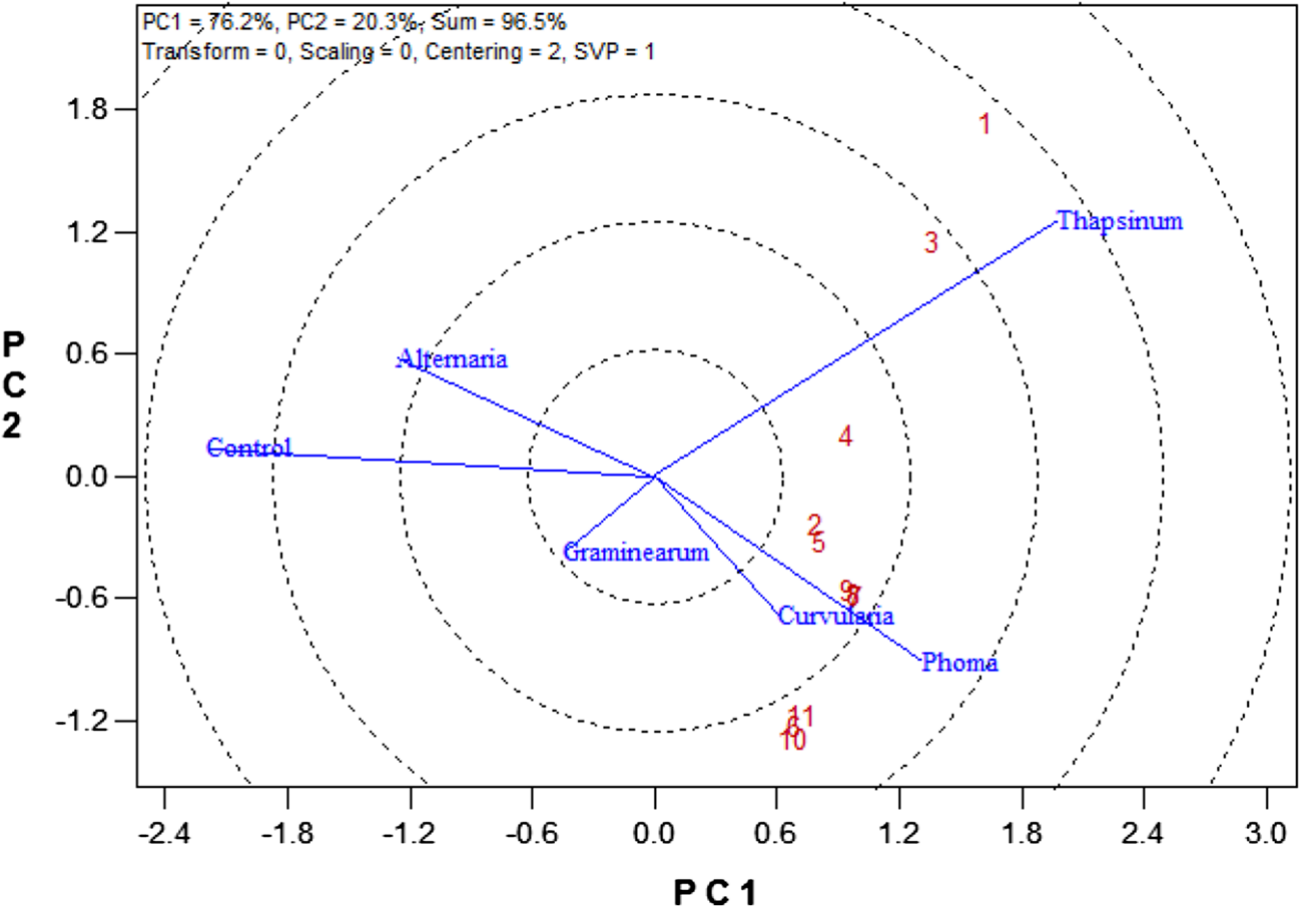
Relationship among the 5 fungal strains (*Fusarium graminearum, Fusarium thapsinum, Curvularia lunata, Phoma* *sorghina* and *Alternaria alternata*) plus a control according to their virulence to the 11 genotypes

Figure 5 shows the relationship among fungal strains. When the angle between vectors connecting two fungal strains is acute, it shows relationship between their behaviors. On the other hand, when the angle between the two vectors is obtuse, it shows a negative relationship. The negative relationship and/or strong crossover genotype-by-pathogen interaction increases as the obtuse angle gets wider. A right angle means there is no relationship in behavior. As such, *Curvularia lunata* and *Phoma* *sorghina* behave similarly. *Alternaria alternata* and the control are in one group. The position of *Fusarium graminearum* places it in between the control and *Phoma* *sorghina*, i.e., causes disease but on a low scale. *Fusarium thapsinum* shows a very weak relation to *Phoma* *sorghina* and shows a negative relation and or strong crossover genotype–pathogen interaction with the rest. *Curvularia lunata* does not relate to *Fusarium thapsinum* and *Fusarium graminearum.* These observations concur with above analysis.

**Fig. 6:**
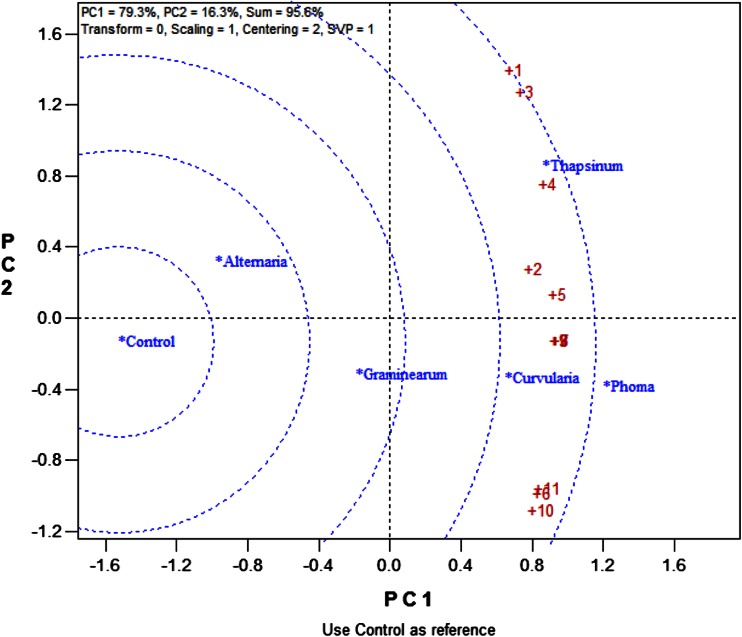
Ranking of the 5 fungal strains (*Fusarium graminearum, Fusarium thapsinum, Curvularia lunata, Phoma* *sorghina* and *Alternaria alternata*) against the control according to their virulence to the 11 genotypes

Figure 6 ranks fungal strain with reference to the control. *Phoma* *sorghina* > *Fusarium thapsinum* > *Curvularia lunata…* > control is the order by which fungal strain ergosterol accumulation is ranked.

### Ranking of genotypes

**Fig. 7:**
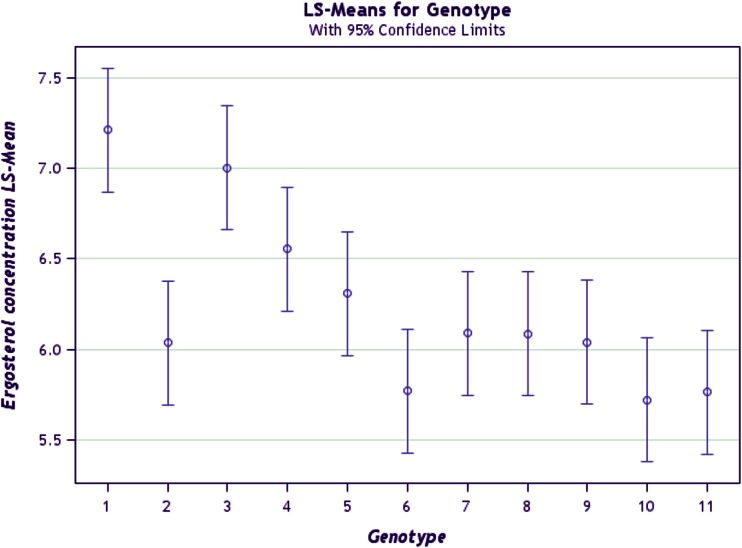
Separation of means for ergosterol concentration for the 11 genotypes for all pathogens

Figure 7 shows how much fungal biomass each of the 11 genotypes accumulated relative to each other. Some of the groupings of genotypes can be gleaned from this figure.

**Fig. 8:**
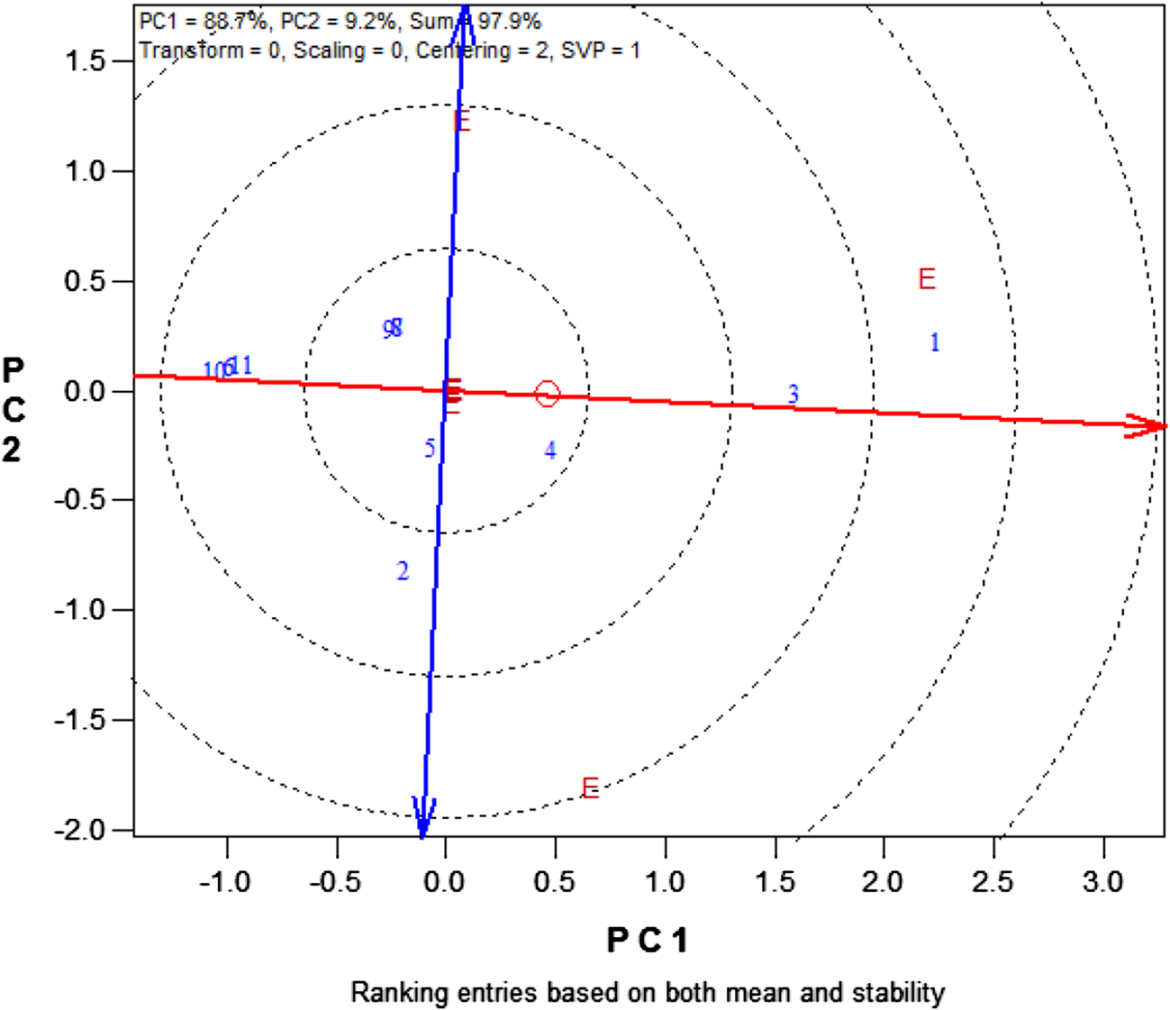
The genotype coordination view to rank genotypes relative to a typical stable genotype (*center* of the concentric circles)

**Fig. 9:**
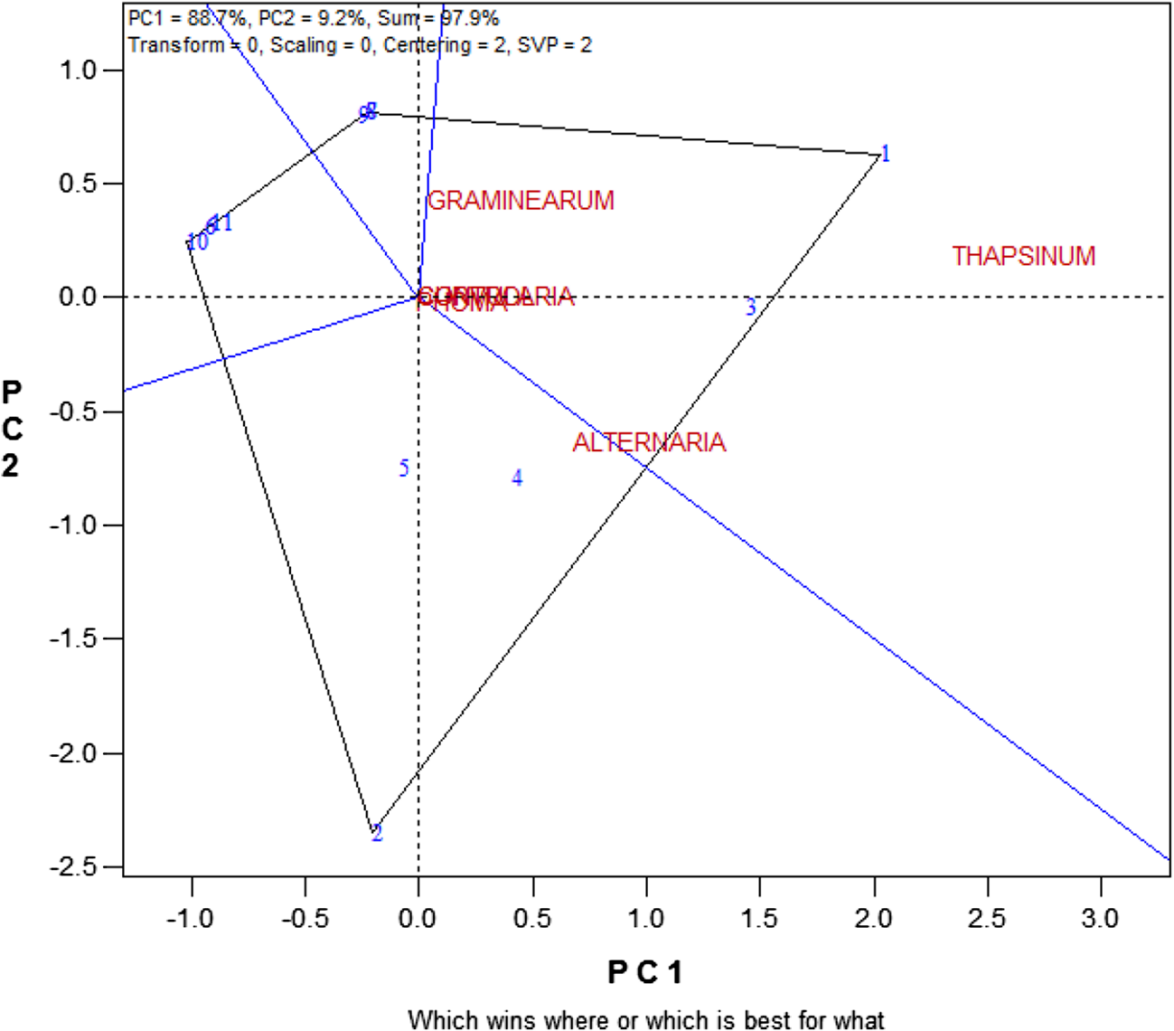
Genotype-focused GGE biplot based on mean ergosterol concentration (μg/g of grain) of 11 sorghum varieties inoculated with spores of 5 fungal isolates (*Fusarium graminearum, Fusarium thapsinum, Curvularia lunata, Phoma* *sorghina* and *Alternaria alternata*) plus a control. The genotypes were used as entries (*blue numeric*) and fungal strains as testers (*red*)

**Fig. 10:**
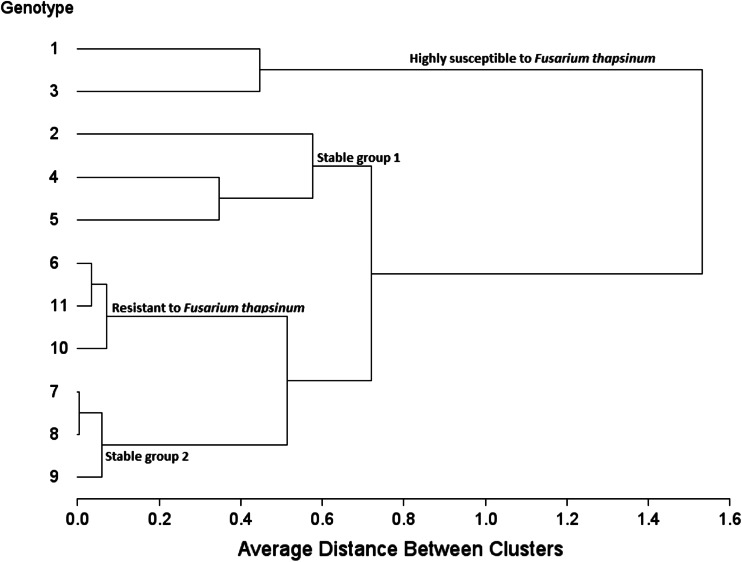
Dendrogram showing clusters of genotypes based on their differential resistance/susceptibility to the five fungal isolates

Figure 8 ranks genotypes along the average tester axis (red line) with arrow pointing to greater mean ergosterol content. The blue line separates genotypes with below average mean ergosterol content from those with above average means. Therefore, genotypes 1 and 3 had the highest mean ergosterol content (upper side of red arrow) due to being highly susceptible to *Fusarium thapsinum*. Genotypes 6, 10 and 11 have the lowest mean ergosterol contents (lower side of red arrow) and are the only group with some resistance genes (resistant to *Fusarium thapsinum* Figs. 9, 10). However, despite having the lowest mean ergosterol contents and showing resistance to *Fusarium thapsinum*, genotypes 6, 10 and 11 were consistently susceptible to *Phoma* *sorghina* (indicating vertical/specific resistance). Specificity of response of genotypes 6, 10 and 11 should be distinguished from stability. This specificity indicates consistence more than stability.

A longer projection to the blue line (Fig. 8), regardless of the direction, represents a greater tendency of genotype-by-fungal strain interaction of a genotype, which means more variable and less stable across fungal strains. A stable genotype in this analysis expresses no genotype-by-fungal strain interaction. Its ergosterol concentration is stable across all fungal strains. Figure 8 shows a typical stable genotype as the little red circle in the concentric circles. Therefore, genotypes closer to the typical stable genotype are the most stable. Genotypes 2, 4 and 5 (stable group 1) are in the same group of more stable varieties since they are on the same side of the red line while on the other hand genotypes 7, 8 and 9 (stable group 2) are in another group of more stable varieties. The stability of stable group 2 genotypes is on the susceptible side.

Figure 9 confirms the grouping obtained in Fig. 8. It looks at “Which won where”, which is an extended use of the “pairwise comparison” function (Yan and Tinker 2006). This figure also emphasizes that genotype 1 was most susceptible to *Fusarium thapsinum* more than any other fungal strains and is in the same group/sector with genotype 3. Genotype 2 is the vertex in the same group with genotypes 4 and 5. Genotypes 6, 10 and 11 are in the same sector. Genotypes 7, 8, and 9 are also in a sector of their own.

Figure 10 is a cluster analysis that confirms the genotype pattern groupings obtained using GGE Biplot analysis (Figs. 8, 9). Both GGE Biplot analysis and Cluster analysis give complementing outputs for both genotype and fungal strain groupings.

## Discussion

The use of ergosterol concentration as a measure of fungal biomass accumulation in sorghum grain has helped to elucidate the intricate host-by-pathogen relations in the grain mold disease. Previous research has identified a wide range of morphological, physical and biochemical factors that confer resistance to grain mold in sorghum. Some of these include grain hardness (Kumari et al. 1992; Mukuru 1992). Jambunathan et al. (1992) demonstrated that ergosterol concentration was negatively and significantly related with hardness values in resistant white sorghum without a testa. Pericarp thickness and pigmentation have been shown to be important traits in resistance to grain molds in maize (Hoenisch and Davis 1994) and sorghum (Singh and Agarwal 1993; Hiremath et al. 1993). Resistance of grains with colored pericarp appears to be due to their phenol content, mainly flavan-4-ols (Martinez et al. 1994). Most of this previous work did not focus on the resistance traits against specific fungi but against the “grain mold complex” fungi. This implies that the observed resistance was dependent on the predominant fungi in the test environments but that was not determined.

It is clear from these results that some of the fungi that are traditionally classified as part of the “grain mold complex” do not cause significant damage or play a major role in the disease complex. Due to highly significant genotype-by-pathogen interactions, it is important to understand which fungal strains cause damage to which sorghum genotypes. *Phoma* *sorghina* and *Curvularia lunata* have been shown to cause significant damage, i.e., significant fungal biomass accumulation across all genotypes whereas *Fusarium thapsinum* shows significant genotype specificity. *Alternaria alternata* on the other hand does not cause much physical damage because it had very little fungal biomass accumulation across all genotypes. The most significant damage caused by *Alternaria alternata* is the production of mycotoxins that are potential food contaminants (Seitz et al. 1975; Jewers and John 1990). *Fusarium graminearum* causes very little grain mold disease. On the side of host genotype, some varieties show vertical/specific resistance even to virulent pathogen strains. The fact that in this group of genotypes used for this experiment, none was resistant to all fungal strains should not be confused to mean that there are no varieties with resistance to *Phoma* *sorghina* and *Curvularia lunata*. If a wider range of genotypes is used, there is potential to find some varieties resistant to *Phoma* *sorghina* and *Curvularia lunata*. This means that all sorghum breeding programs should first of all identify all predominant grain mold associated fungal strains in their areas and then screen their varieties for resistance to those. Specific resistance genes can be tagged with molecular markers and incorporated into commercial varieties so that they have resistance to all major grain mold fungi in their localities.

Grain mold disease scoring should, therefore, not be limited to visual scoring only. This could lead to improper scoring and classification of varieties in response to infection. Failure to properly classify varieties could lead to failure to pyramid resistance genes in susceptible varieties. The only challenge this approach presents is the need for institutions to invest in high throughput HPLC equipment that would enable fast ergosterol analysis so as to analyze many samples during the variety screening process.

## Conclusion

There is significant genotype-by-pathogen interaction in the sorghum grain mold disease complex development. Measurement of ergosterol concentration is a very useful method of assessing the accumulation of fungal biomass once the identity of the causal strain has been ascertained. This way complications and errors associated with visual scoring are easily avoided. Specificity of virulence by the fungal strains and host resistance by the various sorghum plant genotypes makes it prudent for breeding programs to identify prevalent fungal strains in their localities and use that information to screen potential new varieties for resistance genes. Susceptible varieties can then be used in backcross programs with sources of resistance to incorporate or pyramid resistance genes into their genetic backgrounds.
